# Case Report: Feline adrenal pheochromocytoma with a synaptophysin-positive, chromogranin A–negative immunophenotype

**DOI:** 10.3389/fvets.2026.1801002

**Published:** 2026-04-21

**Authors:** Jihyun Kim, Sooa Yoon, Seungjin Lee

**Affiliations:** Lee Seungjin Animal Medical Center, Ulsan, Republic of Korea

**Keywords:** adrenal neoplasia, chromogranin A, feline, pheochromocytoma, synaptophysin

## Abstract

Pheochromocytoma is a rare adrenal medullary neoplasm in cats, with limited published cases and diagnostic challenges due to variable clinical presentation. An 8-year-old neutered male domestic shorthair cat presented with severe watery diarrhea, lethargy, and anorexia. Diagnostic evaluation revealed chronic kidney disease, pyelonephritis, and a large cystic right adrenal mass identified on ultrasonography and computed tomography. Endocrine testing did not support hyperadrenocorticism or hyperaldosteronism, and functional assessment of catecholamine excess was not performed. Surgical adrenalectomy was elected due to mass size and rupture risk. Histopathological examination demonstrated a medullary adrenal neoplasm composed of polygonal cells arranged in characteristic packets. Immunohistochemistry revealed synaptophysin positivity with negative chromogranin A staining, supporting a diagnosis of pheochromocytoma. Postoperative recovery was uneventful, and the cat remained normotensive and clinically stable at one year, with chronic kidney disease managed medically. This case underscores the diagnostic complexity of feline pheochromocytoma and highlights the importance of integrating imaging, histopathology, and immunohistochemistry, as chromogranin A negativity does not exclude this diagnosis.

## 1 Introduction

Pheochromocytoma is a catecholamine-secreting neuroendocrine tumor that originates from the chromaffin cells of the adrenal medulla (1). Pheochromocytoma is rare in cats and has been reported only in a limited number of case reports (2–8). This report describes the diagnostic workup, computed tomography (CT) findings, surgical management, histopathological and immunohistochemical features, and clinical outcomes of a case of feline pheochromocytoma.

## 2 Case description

**Figure 1:**
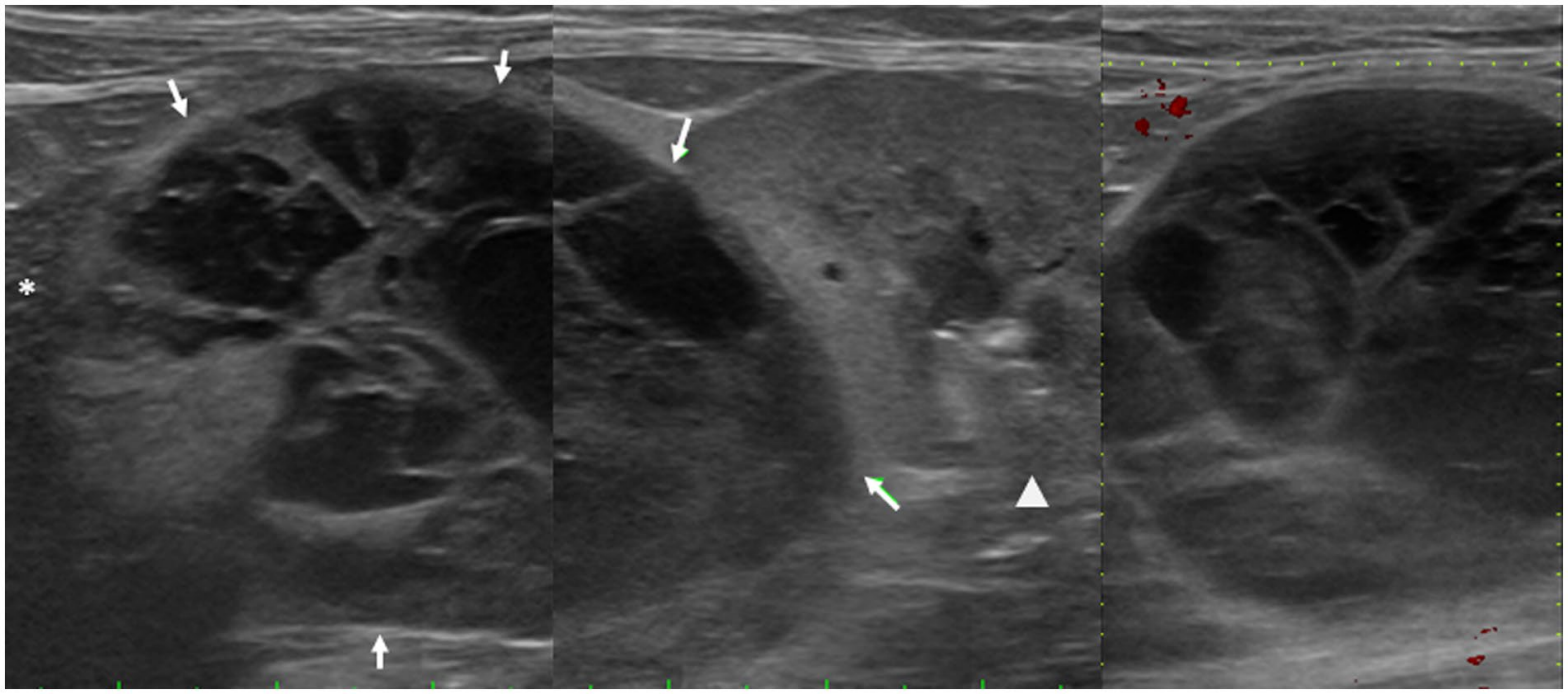
Abdominal ultrasonographic image of a cat with adrenal pheochromocytoma. A well-marginated, multiseptated cystic structure (arrows) is observed in the retroperitoneal space between the caudate process of the liver (asterisk) and the right kidney (arrowhead). The mass is surrounded by a thin capsule and demonstrates sharp margins with clearly distinguishable boundaries from both the caudate process and the right kidney, raising suspicion of adrenal origin. No intralesional blood flow is detected on Doppler examination.

**Figure 2:**
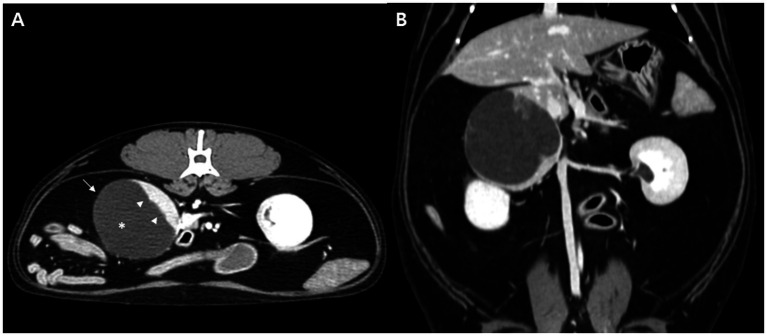
Dynamic computed tomography images (delayed phase) of a cat with adrenal pheochromocytoma. Axial **(A)** and dorsal **(B)** plane images demonstrate a thin-walled cystic structure within the right adrenal gland (arrow). Marked crescent-shaped peripheral contrast enhancement is observed along the margin of the lesion (arrowhead), accompanied by mild enhancement of the cyst wall. The lesion is predominantly filled with fluid attenuation material (asterisk).

An 8-year-old neutered male domestic shorthair cat presented with severe watery diarrhea (fecal score of 7), lethargy, and anorexia. Fecal examination, complete blood count, serum biochemistry, and blood gas analyses were performed; all results were within normal limits except for a mildly elevated white blood cell count. SBP (systolic blood pressure), measured using oscillometry, was 140 mmHg, which falls within the borderline range according to ACVIM guidelines (9). Abdominal ultrasonography revealed bilateral renal cortical hyperechogenicity with mild loss of corticomedullary distinction, consistent with chronic kidney disease, as well as bilateral renal pelvic dilatation accompanied by increased echogenicity of the perirenal retroperitoneal fat, which was suggestive of pyelonephritis. In addition, a well-marginated, multiseptated cystic mass measuring 4.6 × 4.4 cm was identified in the retroperitoneal space between the caudate process of the liver and the right kidney, consistent with an adrenal origin (Figure 1). Urinalysis revealed hyposthenuria (urine specific gravity, USG = 1.009) and a urine protein-to-creatinine (UPC) ratio of 1.5. Urine culture revealed *Acinetobacter pittii*, and antibiotic therapy was initiated to control pyelonephritis. CT revealed a thin-walled cystic lesion in the right adrenal gland with crescent-shaped peripheral enhancement and fluid-filled contents (Figure 2). The contralateral adrenal gland appeared normal in terms of size and architecture. Thoracic CT revealed no abnormalities. A two-month course of antibiotics was administered to treat pyelonephritis, resulting in stable management of chronic kidney disease at IRIS stage 1 with normotension.

An endocrine investigation was conducted to determine whether the adrenal tumor was functional and to predict its most likely origin. An adrenocorticotropic hormone (ACTH) stimulation test was performed. Basal serum cortisol concentration was 2.2 μg/dL (reference interval, 1–6 μg/dL), and post-ACTH cortisol concentration was 6.1 μg/dL (reference interval, 8–18 μg/dL). The ACTH stimulation test results did not support a diagnosis of hyperadrenocorticism (HAC), although the condition could not be definitively excluded. Plasma aldosterone measured 1.5 (reference interval, 7–14). Plasma and urinary metanephrine and normetanephrine concentrations were not measured.

Although the functional status and hormonal origin of the adrenal mass could not be definitively determined, surgical excision was elected due to the large size of the mass and concern for potential rupture. The preoperative SBP was 170 mmHg, consistent with systemic hypertension; however, stress-related elevation could not be excluded because sustained hypertension was not documented. Prothrombin time and activated partial thromboplastin time values were within the reference intervals. Phenoxybenzamine was not administered preoperatively.

Surgical adrenalectomy was performed, and the adrenal mass was removed without evidence of local invasion or adhesion. Intraoperative SBP remained stable (approximately 80–120 mmHg), and heart rate was maintained at approximately 160 beats per minute; no intraoperative complications were observed. Postoperative recovery was uneventful. SBP was monitored every 12 h, and blood urea nitrogen, creatinine, and electrolyte levels were evaluated every 24 h; all parameters remained within reference ranges. The cat was discharged on postoperative day 3.

**Figure 3:**
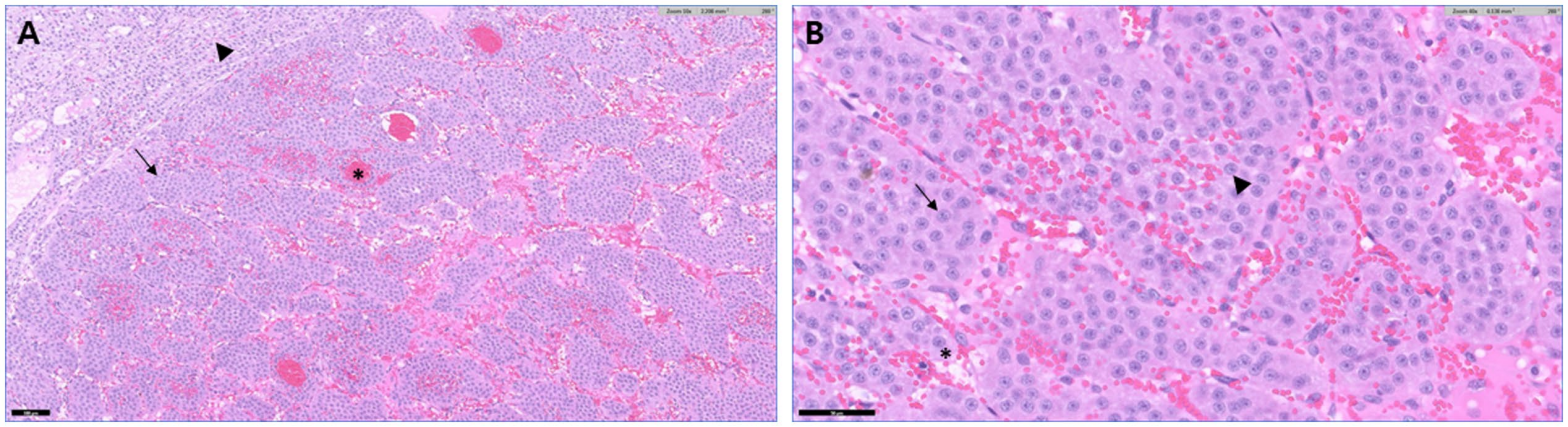
Histopathological features of the adrenal pheochromocytoma (hematoxylin and eosin stain). **(A)** Junction between the pheochromocytoma (arrow) and the adrenal cortex (arrowhead), with neoplastic cells arranged in characteristic packets separated by fine fibrovascular stroma and areas of hemorrhage (asterisks) (×10; scale bar = 100 μm). **(B)** Polygonal neoplastic cells (arrow) with moderate amounts of eosinophilic cytoplasm (asterisk) and round nuclei (arrowhead) containing a small nucleolus (×40; scale bar = 50 μm).

**Figure 4:**
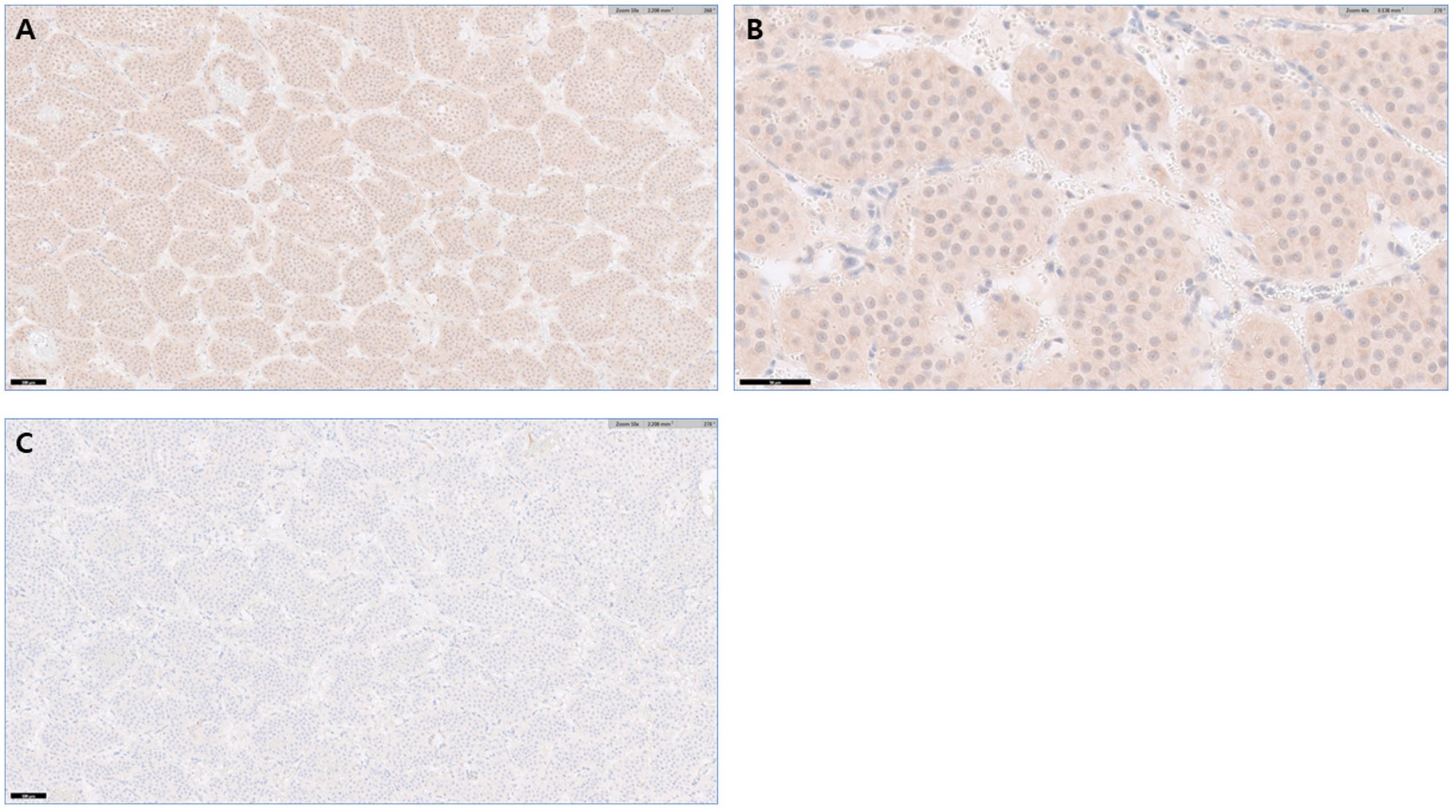
Immunohistochemical features of the adrenal pheochromocytoma. **(A)** Synaptophysin immunohistochemistry showing mild cytoplasmic immunoreactivity in neoplastic cells (×10; scale bar = 100 μm). **(B)** Higher magnification of synaptophysin staining demonstrating mild cytoplasmic immunoreactivity of neoplastic cells (×40; scale bar = 50 μm). **(C)** Chromogranin A immunohistochemistry showing absence of immunoreactivity in neoplastic cells (×10; scale bar = 100 μm).

Histopathological and immunohistochemical examinations confirmed the diagnosis of pheochromocytoma. Histopathology revealed an adrenal medullary mass expanding and compressing the adjacent cortex. The neoplasm was composed of polygonal cells arranged in characteristic packets separated by fine fibrovascular stroma with multifocal hemorrhage. The neoplastic cells contained moderate amounts of eosinophilic cytoplasm and round nuclei with small nucleoli (Figure 3). Immunohistochemistry was performed at a commercial reference laboratory (IDEXX Laboratories) using a polyclonal rabbit anti-synaptophysin antibody (BioSB, catalog no. BSB 5950) and a polyclonal rabbit anti-chromogranin A antibody (Abcam, catalog no. ab45179) at the manufacturer-recommended dilutions. Appropriate positive and negative controls were included in each staining run. Pancreatic tissue served as the control tissue; sections incubated with the primary antibody served as positive controls, whereas sections processed with omission of the primary antibody served as negative controls. Control tissues demonstrated appropriate immunoreactivity, confirming staining adequacy. The neoplastic cells showed mild cytoplasmic immunoreactivity for synaptophysin and were negative for chromogranin A (Figure 4).

One year postoperatively, the cat remains normotensive and clinically stable under the management of IRIS stage 2 chronic kidney disease and proteinuria.

## 3 Discussion

Pheochromocytoma remains a rare adrenal medullary tumor in cats, with only a small number of histopathologically confirmed cases reported to date (2, 3, 5, 6, 8). This report describes a stepwise diagnostic approach to feline pheochromocytoma, integrating clinical presentation, diagnostic imaging, histopathology, and immunohistochemistry to achieve a definitive diagnosis.

Immunohistochemical staining using the adrenomedullary markers synaptophysin and chromogranin A has been described in only two previously reported feline cases. These include one case of pheochromocytoma (8) and another case in which an adrenocortical adenoma was diagnosed in an adrenal gland with a pheochromocytoma in the contralateral gland (5), where neoplastic cells showed positive immunoreactivity for synaptophysin and chromogranin A.

In the present case, histopathological examination revealed a neoplasm composed of polygonal cells arranged in characteristic packets separated by fine fibrovascular stroma with associated hemorrhage. Immunohistochemical findings, including mild cytoplasmic synaptophysin immunoreactivity and negative chromogranin A staining, support an adrenal medullary origin and are consistent with a diagnosis of feline pheochromocytoma.

Large-scale immunohistochemical analyses have demonstrated that neuroendocrine marker expression varies across tumor types and that chromogranin A may be absent, despite synaptophysin positivity in humans. In a human cohort of 48 pheochromocytoma cases, one case (2.1%) showed negative chromogranin A staining and positive synaptophysin expression, whereas the remaining 47 cases (97.9%) were positive for both markers (10). To the best of our knowledge, this represents the first reported case of feline pheochromocytoma characterized by synaptophysin positivity in the absence of chromogranin A immunoreactivity.

Functional assessment of adrenal tumors in cats remains challenging. Biochemical confirmation of catecholamine excess using plasma or urine metanephrine and normetanephrine concentrations may aid in the diagnosis of pheochromocytoma (11). These assays were not performed in the present case because they are not routinely available in our region. Consequently, preoperative diagnostic certainty was limited.

Differential diagnoses for a cystic adrenal lesion in cats include adrenal hemorrhage, non-neoplastic cysts, and cystic degeneration of adrenal tumors. Fine needle aspiration (FNA) with cytological evaluation has been proposed as a minimally invasive diagnostic tool to differentiate adrenal cortical and medullary tumors in dogs, with moderate diagnostic accuracy and a low complication rate (12). However, in cats, the diagnostic utility of FNA for adrenal masses remains unclear, and the procedure may carry potential risks, including hemorrhage or catecholamine-associated complications. Therefore, FNA was not performed in the present case, and definitive diagnosis relied on histopathological and immunohistochemical evaluation following surgical excision.

The sensitivity of the ACTH stimulation test for the diagnosis of HAC in cats has been reported to be only 56% (13); therefore, this test is not recommended as an initial diagnostic screening tool for cats with suspected HAC. Accordingly, a low-dose dexamethasone suppression test (LDDST) is generally preferred for the diagnosis of HAC. However, in the present case, the ACTH stimulation test was selected to shorten the diagnostic time following consultation with the owner because the cat exhibited marked stress associated with hospitalization. In addition, concurrent pyelonephritis precluded the use of dexamethasone and limited the feasibility of LDDST.

In the present case, aldosterone and post-ACTH cortisol concentrations were below the reference intervals (13, 14). Although adrenal medullary neoplasia may have contributed to secondary adrenocortical suppression, alternative explanations include chronic systemic illness, assay variability, and physiologic stress. Postoperative adrenal function was not formally reassessed; however, no clinical signs of hypoadrenocorticism were observed during follow-up.

In dogs with pheochromocytomas, the preoperative administration of phenoxybenzamine has historically been recommended to reduce perioperative complications. A previous study reported that preoperative phenoxybenzamine administration reduced postoperative mortality from 48 to 13% (15). However, more recent studies have reported conflicting results. Two subsequent studies demonstrated that preoperative phenoxybenzamine administration did not significantly improve perioperative outcomes or survival in dogs undergoing adrenalectomy for pheochromocytoma (16, 17). To date, no studies have evaluated the effects of preoperative phenoxybenzamine administration in cats undergoing adrenal surgery.

In this case, transient preoperative elevations in systolic blood pressure were observed, but sustained hypertension was not documented. Accordingly, prophylactic phenoxybenzamine was not administered. Blood pressure remained stable intraoperatively and postoperatively, and measurement variability was likely attributable to stress-related hypertension.

## 4 Conclusion

To our knowledge, synaptophysin positivity in the absence of chromogranin A immunoreactivity has not previously been documented in feline pheochromocytoma. This case highlights the diagnostic complexity of feline pheochromocytoma and underscores the importance of integrating histopathology and immunohistochemistry for accurate diagnosis.

## Data Availability

The original contributions presented in the study are included in the article/supplementary material, further inquiries can be directed to the corresponding author/s.
